# Draft genome sequence of basidiomycete yeast *Rhodotorula mucilaginosa* DH4148 from a patient with fungemia

**DOI:** 10.1128/mra.00821-26

**Published:** 2026-09-25

**Authors:** Juhita Dhar, Prince B. Amoah, Nicole A. Loeven, Deborah A. Hogan, Jason E. Stajich

**Affiliations:** 1 Department of Microbiology and Plant Pathology, University of California, Riverside 8790, Riverside, California, USA; 2 Institute for Integrative Genome Biology, University of California, Riverside 8790, Riverside, California, USA; 3 Department of Pathology and Laboratory Medicine, Dartmouth Hitchcock Medical Center 22916, Lebanon, New Hampshire, USA; 4 Department of Microbiology and Immunology, Geisel School of Medicine at Dartmouth, Hanover, New Hampshire, USA; Rochester Institute of Technology, Rochester, New York, USA

**Keywords:** *Rhodotorula*, fungemia, yeast, genomics

## Abstract

*Rhodotorula mucilaginosa* is a basidiomycete yeast with ecological and emerging clinical relevance. Here, we report a high-quality *de novo* genome assembly of *R. mucilaginosa* strain DH4148 isolated from a patient with fungemia.

## ANNOUNCEMENT

*Rhodotorula mucilaginosa* is a pigmented basidiomycetous yeast that is globally distributed across aquatic, terrestrial, and built environments. The fungus is a frequent human commensal and can occur as an opportunistic pathogen with strain-specific virulence in immunocompromised hosts (1–4).

**Fig 1:**
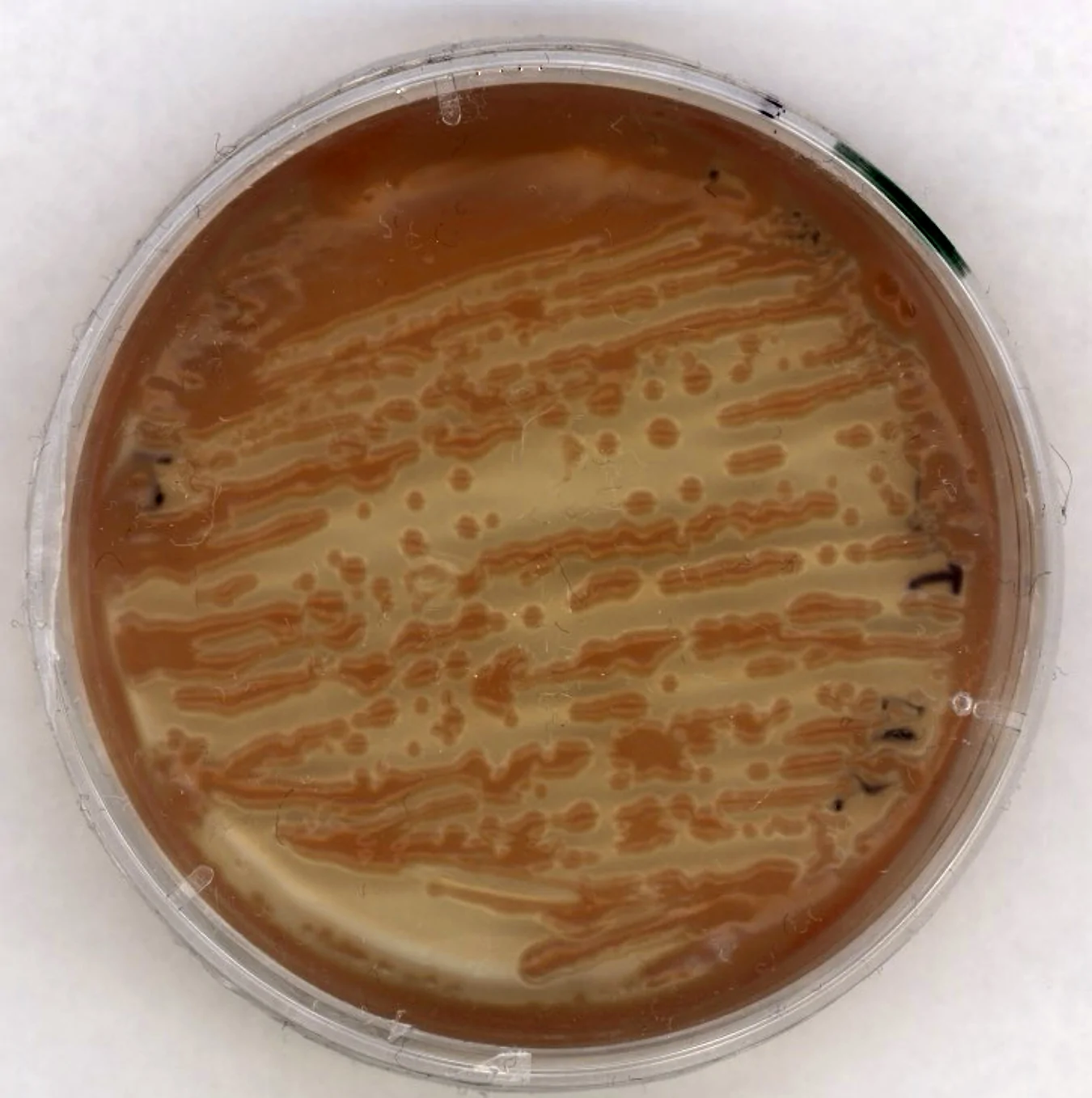
*R. mucilaginosa* strain DH4148 growing on YPD agar.

The strain originated from venipuncture blood incubated in BACTEC FX (BD, Franklin Lakes, NJ) automated blood culture instrument for 45 h at 35°C ± 1.5°C. Yeast cells were observed by Gram stain in a positive culture, incubated for 3 days at 35°C on Sabouraud Dextrose agar and identified as *Rhodotorula mucilaginosa* by MALDI-TOF MS (Clinical Microbiology Laboratory, Dartmouth Hitchcock Medical Center). Following clinical protocol 00020786, the sub-cultured isolate was accessioned as DH4148 without patient identifying information. The isolate was transferred to UC Riverside and stored at −20°C in Yeast Peptone Dextrose (YPD) broth with glycerol. DNA was obtained from a streaked culture grown on YPD agar (10 g/L yeast extract, 20 g/L peptone, and 20 g/L dextrose) and incubated at 27°C for 48 h (Fig. 1). Scraped cells were processed into liquid nitrogen and DNA extracted with Wizard High Molecular Weight kit (Promega Corporation, Madison, WI); purity, integrity, and concentration were assessed by NanoDrop (NanoDrop Technologies, Wilmington, DE), agarose gel electrophoresis, and Qubit (Invitrogen, Carlsbad, CA).

Extracted DNA was shipped to SeqCoast (Portsmouth, NH, USA) for whole-genome library preparation and sequencing. Short-read libraries were constructed with Illumina DNA Prep tagmentation kit (#20060059) with Unique Dual Indexes and sequenced on the Illumina NextSeq2000 platform (Illumina Inc, San Diego, CA) on a 300-cycle XLEAP-SBS flow cell kit paired 2 × 150 bp format. Read demultiplexing, trimming, and analytics were prepared using DRAGEN v4.2.7 (Illumina, Inc). Long-read libraries were prepared with the Oxford Nanopore Technologies (ONT) Native Barcoding Kit (#SQK-NBD114) and Long Fragment Buffer. The library was sequenced on PromethION 2 Solo platform (FLO-PRO114M Flow Cell, vR10.4.1) with a translocation speed of 400 bp. Base calling was performed with the super-accurate model and barcode trimming using MinKNOW v24.11.8 and Dorado v7.6.7 (Oxford Nanopore Technologies, Oxford, UK).

The ONT reads were assembled with Flye v2.9-b1774 (5) (-nano-hq --scaffold, default parameters otherwise) and polished with Medaka v2.1.1 (Oxford Nanopore Technologies, Oxford, UK). Further polishing with short-read by POLCA v4.1.4 (6) using short reads run within the AAFTF v0.6.0 pipeline (7). Assembly contigs were scaffolded with RagTag v2.1.0 (8) and reference strain genome GCA_034406715.1. Telomeric sequences were identified using find_telomere.py, and repeats were masked using RepeatModeler v2.0.6 (9) and RepeatMasker v4.1.8 (10) incorporating fungi from RepBase (11). Assembly statistics and completeness were assessed with QUAST v5.3.0 (12) and BUSCO v5.4.3 (13) using the basidiomycota_odb10 marker set (Table 1). Assembly consistency was confirmed by aligning raw reads using minimap2 v2.30 (14) for ONT reads and BWA MEM v0.7.19 (15) for Illumina. Mapping statistics were calculated with samtools v1.22.1 flagstat (16) and depth of coverage with mosdepth v0.3.3 (17).

**TABLE 1:** The genome sequencing, assembly, and annotation statistics of *R. mucilaginosa* DH4148

Statistic	Values
Total assembly size (Mb)	20.37
Number of contigs	37
L50	7
L90	15
N50 (bp)	1,299,790
N90 (bp)	685,277
Largest contig (bp)	2,165,634
Total Illumina reads	3,981,848
Total ONT reads	4,140,446
Total ONT sequence (Mb)	4,212
Raw ONT read N50 (kb)	2.1
Illumina mean coverage	26.54×
ONT mean coverage	200.86×
Illumina mapping rate	98.17%
ONT mapping rate	96.67%
GC content (%)	60.48
tRNA genes	135
Predicted protein-coding genes	6,773
Predicted mRNA transcripts	7,290
Alternatively spliced genes (>1 isoform)	424
BUSCO completeness	97.3% (1,715 markers)
BUSCO single copy	91.7% (1,712 markers)
BUSCO duplication	0.2% (3 markers)
BUSCO fragmented	0.3% (5 markers)
BUSCO missing	2.4% (44 markers)

The assembly shared 99% average nucleotide identity to *R. mucilaginosa* genomes in NCBI with skani v0.3.2 (18). Gene prediction with Funannotate v1.8.17 (19) proceeded with “train” using public RNA-Seq data, trimmed with Trimmomatic v0.39 (20), assembled with Genome-Guided Trinity v2.15.1 (21), and aligned with PASA v2.5.2 (22) for Augustus v3.5.0 (23) training evidence. Funannotate “predict” built exon supporting evidence gene prediction using Diamond v2.1.8 BLASTX (24) and Exonerate v2.4.0 (25) of Swiss-Prot proteins (26). Composite gene models from Augustus, SNAP v (27), GlimmerHMM v3.0.4 (28), and GeneMark-ES/ET v4.72 (29) predictions were made by EVM v1.1.1 (22). The tRNA genes were predicted with tRNAscan-SE v2.0.12 (30). Funannotate “update” added untranslated regions (UTRs) and alternative splicing with PASA (22).

Funannotate “annotate” identified protein domains, sequence similarities, and functional classification with Pfam v37.3, InterProScan 5.75-106.0 (31, 32), UniProt v2025_02 (26), and eggNOG-mapper v2.1.12 (33, 34). Peptidase and carbohydrate-active enzymes were identified using MEROPS v12.5 (35) and dbCAN v11.0 (36). AntiSMASH v7.0 (37) predicted one non-ribosomal peptide synthetase (NRPS), one NRPS-like, and three terpene synthase biosynthetic gene clusters.

## Data Availability

This whole-genome shotgun project has been deposited at DDBJ/ENA/GenBank under the accession number JBZGVR000000000. The version described in this paper is JBZGVR010000000, and the assembly under accession number GCA_058775505.1. Sequence reads were deposited under SRA project accession numbers SRR37106098 (ONT), SRR37106099 (Illumina), BioProject accession number PRJNA1418719, and BioSample accession number SAMN55059426. Public RNA-seq from NCBI SRA database was used for gene training and annotation support project: SRP107244, SRP233021, SRP235675, SRP258902, and SRP279652. Genomes compared for ANI included GCA_012922695.1.
